# Buffalo Pathological Society

**Published:** 1890-04

**Authors:** Eugene A. Smith

**Affiliations:** Secretary


### THE BUFFALO PATHOLOGICAL SOCIETY.

Reported by EUGENE A. SMITH, M. D., *Secretary*.

REGULAR meeting, February 21, 1890, the president, Dr. DELANCEY ROCHESTER, in the chair.

The subject for the evening was Tumor Albus, and Dr. S. Y. HOWELL read the first paper on

### PATHOGENESIS AND MORBID ANATOMY.

From the earliest times down to the present, a very fair share of medical literature has been devoted to the consideration of a chronic form of disease, attacking chiefly the diarthrodial or movable joints, and deriving its importance from the fact that the most distressing deformities and even a lingering, miserable death were but too frequently its ultimate outcome. Children were found to be the favored victims, though even the aged were not exempt; and the hip and knee were the articulations most often involved.

A further peculiarity of the disease rested in the fact that a joint thus affected exhibited but in part the symptoms which were deemed cardinal in inflammatory conditions. Instead of being hot and red, the joint was cool, pale, smooth and shiny; swelling, however, was present, together, perhaps, with impairment of function and pain. We cannot wonder, then, that as early as 1734, Wisemann first applied to this condition the simple but expressive term, "Tumor Albus," or "White Swelling," the tumeur blanche of the French, with which we are all so familiar. As used by Wisemann, however, the name of the disease referred merely to its surface indications, but, as careful dissections multiplied, as the microscope revealed histological details, and as pathology advanced, many new terms were employed. Indeed, the nomenclature of this form of arthritis affords one a very fair picture of the evolution of modern medicine from its primitive simplicity. Thus, Sir. Benj. Brodie replaced "tumor albus" with "pulypy degeneration of the synovial membrane;" Barwell, with "strumous synovitis or arthritis;" Velpeau, Bonnet and Richet, with "tumeur fongeuse;" Billroth and Volkmann, with "Tuberculöse Gelenkentzündung," or "fungous arthritis;" Hütter, with "synovitis hyperpatica fungosa and pannosa." Among other synonyms we have tuberculous synovitis, granuliorinde tuberculöse Gelenkentzünding, pyarthrosis, gliedschwamm, ungus farticuli, empyema articulorum, gelatinous arthritis, and cold abscess of joint.

In pre-antiseptic days, before the dissections on the living subject (*in vivo*) which have now become an old story to us all, were possible, the material for the study of this disease was limited. Structures afforded by post-mortems in subjects who had succumbed to tuberculosis, amyloid changes, or marasmus, together with parts removed by amputation or possibly resections, in the latest stages of the malady, were alone available. It is no wonder, then, that tumor albus was formerly believed to be a diffused process, due to diffuse inflammations, gradually progressing, and leading ultimately, in the worst cases, to suppuration and destruction of joints. In these cases the synovial membrane and adjacent capsule were always found markedly diseased. The inner surface of the joint had been converted into a peculiar, thick, jelly-like, "fungous" layer of granulation tissue, outside of which lay an extensive stratum of gelatinous, edematous, or more fibrous structures, which, especially in the knee, hip and elbow, involved even the skin, thus completely enveloping the joint. Often, however, when the soft parts were thus extensively disorganized the articular cartilages were found to be little if at all affected, a condition of affairs which justified the commonly accepted belief, that in the great majority of cases the disease began in the synovial lining. Even should the cartilages have been destroyed, and the joint ends of the bones more or less disorganized, it was very natural to regard this as secondary to the synovial inflammation and suppuration. The cartilage, bathed in and absorbing the purulent synovial exudates, broke down. Or the granulation tissue advancing from the edges of the cartilage over its surface and remaining in contact with or penetrating it, caused its erosion, perforation, and final reversion into a soft, cellular tissue of a more immature type. The subjacent bone next yielded to the progress of the triumphant granulation tissue and a fungous caries was the result. All these destructive changes had been furthered by the pressure of the joint upon the softened cartilages and inflamed bone.

That the synovial lining of joints was the tissue in which the successive changes resulting in tumor albus began, was apparently supported by other observations. While the etiological relationship of scrofula and tuberculosis to this disease has long been recognized, the existence of a specific virus even though suspected had never been demonstrated. Besides, experience had also shown that almost all the diseases now classed as infectious might be complicated with diffuse inflammation of joints, beginning in the synovial membrane. Pyemia, septicemia, puerperal fever, syphilis, glanders, dysentery, gonorrhea, malaria, whooping cough, epidemic parotitis (mumps), erysipelas, diphtheria, cerebro-spinal meningitis, typhoid and typhus fevers, small-pox, measles and scarlatina—all these were at times attended with or followed by acute synovitis of one or more joints. That the joint complication was an evidence of metastasis, was as little known as were the bacterial agents to which such metastatic inflammations are now ascribed.

Although professional opinion was thus overwhelmingly in favor of the view that fungus arthritis was almost invariably the outcome of a primary synovitis, it was still admitted that occasionally the disease began in bone. The whole epiphysis was at times found to be red and soft; or, again, merely the layers immediately beneath the cartilaginous covering of the joint-surface were thus diseased, the medullary substance being changed into granulation tissue. The cartilage was found more or less eroded, raised, or even perforated by the growing granulations beneath it—conditions which indicated a primary diffuse osteomyelitis extending toward the joint-cavity. This doubt concerning the histogenesis of fungous joint disease, however, is gradually being cleared up by the labors of the modern surgeon and pathologist. Operative interference is resorted to early in joint-troubles, thanks to the confidence inspired by the results of antiseptic precautions, and exploratory dissections *in vivo* are made with comparative impunity. Instead of thoroughly disorganized joints, which were well-nigh the sole available material for the instruction of the surgeon formerly, the latter may now search for and remove the diseased tissues ere the work of destruction has gained great headway. The effect of these opportunities is evidenced by the altered views of to-day. While Billroth claims that fungous disease of the joints attacks primarily the synovial membrane in more than fifty per cent. of the cases, Müller has found in examining 232 preparations, mainly from resections, that only forty-six were of synovial origin, 158 starting in the bony structures. König reports forty-seven out of seventy-one cases as beginning in the bones. The most radical champion of the osseous origin of tumor albus, however, was found in the person of the brilliant Volkmann, whose death we've so recently been called upon to mourn. It was my privilege to witness the work of this talented man almost daily during a period of four months; and if my statements savor strongly of his teachings, I trust that I may be pardoned, since repeated demonstrations have convinced me of their truth. According to Volkmann, the diffuse degenerative changes which we've been considering are *secondary* phenomena. The fungous arthritis begins in *diseased foci*, and it is to the invasion of the joint-cavity by the liquefying contents of these focal points that the epiphyseal and synovial inflammations are due. The danger to the joint, then, becomes imminent when softening of these foci ensues. The foci are generally small, not often exceeding a cherry-stone or hazel-nut in size. They are located in or on the bone, frequently at some distance from the articular cartilage; indeed, they may lie in the shaft of the bone, outside of the epiphyseal cartilage. Such a primary focus represents a very circumscribed cheesy or tubercular ostitis or osteomyelitis, and the subsequent history of the case depends largely upon the fate of this infectious centre. Should it become capsulated, shrink and become absorbed, or should its fluid contents discharge through the skin outside of the joint, specific infection of the latter is often avoided. During this prodromal stage, however, the joint does not always escape. The focal disturbances may be followed by or attended with congestion of the periosteum, synovial membrane, and the other peri-articular tissues. Reactive inflammation ensues; the joint appears swollen and doughy; considerable exudation may occur in the joint-cavity; the synovial membrane is congested, swollen, and assumes the character of dense granulation tissue; while migrated leucocytes may occasion a slight opacity of the increased synovial fluid. The joint-cavity may become obliterated, to a greater or less extent and, in case the irritation is very marked, muscular contraction may ensue and a clinical picture of fungous arthritis present itself, though the process is thus far of an innocent character.

Such changes, severe as they are, may prove conservative. The partial obliteration of the joint and the diminished synovial surface render their subsequent specific infection by the contents of the primary focus less serious. This is often shown in the knee, especially in children. There, when infection does ensue, one encounters those total suppurations of the joint which distend the capsule with pus, and those carious denudations of the joint-surfaces, much less often than in the hip. The process is more of a purulent character in the hip, of a fungous character in the knee.

Hütter, however, has long since shown that similar processes may obtain in the hip-joint. Central foci in the neck, or even in the trochanter, lead *first* to obliteration of the joint-cavity. Then these foci extend, destroying the epiphyseal cartilage; the head separates from the neck, and remains firmly grown to the acetabulum; while the shaft is displaced backward and upward upon the ilium, as occurs in fracture of the neck.

Let us now devote a moment to the focal centres of infection.

In resecting a joint, one recognizes such a focus as a circumscribed, yellowish spot in the spongy tissue, which is anemic, dry, opaque, and slightly cheesy in character. The bone-tissue about the focus is often, but by no means always, of a lively red color, and softened by reactive inflammation; while within the cheesy part the spongy tissue exhibits its normal firmness and density, or even a well-marked sclerosis. Immediately surrounding the yellowish focus the naked eye often detects a number of gray miliary and submiliary granules, which are undoubtedly tubercles. Or, the focus is wholly separated from its environment and forms a small sequestrum, which is entirely surrounded by a thin pale-gray or pale-violet, jelly-like zone of granulation tissue, thickly infiltrated with tubercles.

When of a certain age these tubercles are circumscribed, roundish formations with the familiar reticulum, giant cells toward the centre, the next zone of epithelioid cells, and the whole enveloped by a zone of lymphoid elements. Vessels are absent, and hence proneness to undergo fatty metamorphosis. Usually these tubercular foci are single, or several may occur in the same epiphysis. It is comparatively seldom, however, that more than one of the bones entering into the formation of a joint is thus primarily affected.

The favorite sites of the foci in bone are the olecranon, both condyles of the humerus, calcaneus, internal condyle and neck of femur,—the latter much more frequently so than the head proper. The acetabulum is not infrequently the site of this focal disease (acetabular coxitis).

Contact with the liquid, cheesy masses and the pus, containing the tubercular virus, is always followed by an eruption of miliary tubercles.

In the granulations about the small sequestrum, in the walls of the abscesses which form underneath the periosteum and between the muscles, in the synovial membrane and its communicating cavities, in the fistulous channels through which the pus is evacuated—everywhere, miliary tubercles point the way taken by the specific and deadly microbe. Wherever these secondary granulomata undergo cheesy metamorphosis and disintegrate, the granular debris constitutes new infectious material. These facts account for the obstinacy of the inflammation, its chronicity, and its recurrence after apparent healing or latency.

The location of the focus determines largely the secondary infection of the joint by the pus. The nearer to the joint-cartilage, the capsular insertion, and the synovial pockets communicating with the joint, the easier becomes the invasion of the latter. The attachment of the capsular ligament has an important bearing in this respect. When it is far from the articular surface, as in the upper end of the femur, the probability of a focus discharging its contents within the joint cavity is much greater than in the case of the knee, for example, where the capsular attachment is just beyond the articular surfaces. When intra-articular rupture does occur (and it is well to remember that this is the rule rather than the exception) a diffuse tubercular synovitis is excited, which extends, in the manner already described, to the cartilage and bone; and, sometimes by suppuration, sometimes by the formation of fungous granulation tissue, alters and destroys the whole joint apparatus.

Tubercles are now present everywhere in the diseased joint ; and the fluid containing the products of their disintegration may break through the capsule, and, gravitating along the intermuscular planes and beneath the skin, form tubercular abscesses of variable sizes, or discharge externally.

When operating, the fistulous tracts connecting these various purulent deposits can generally be found. Spontaneous healing may ensue at any stage of the disease. Should this take place before the cartilages are much altered, the usefulness of the joint may be quite restored. After the partial or complete destruction of the cartilage, however, perfect motion is no longer to be looked for, more or less anchylosis being present.

In the late stages, when recovery ensues, the greatest amount of permanent injury remains. The destruction of the joint-surfaces permits luxation to occur, which the muscular contraction tends to confirm and intensify. The granulation tissue ceases to destroy and attempts the work of repair by forming bands of dense cicatricial tissue between the joint surfaces. The resulting immobility is intensified by a similar connective tissue formation in the capsular and other ligamentous structures. Such a fibrous anchylosis may be succeeded by bony anchylosis, in which case the cicatricial bands undergo ossification.

The fact that the granulomata found in tumor albus are genuine tubercles has been abundantly demonstrated by the most competent authorities. Tubercle bacilli, though few in number and difficult to find, are oftenest met with in the giant-cells, though occurring in the other cells of the tubercle as well. They are found more easily in the early stages of the disease.

1. R. Koch found the bacilli in two out of four cases of this disease. Schuchardt and Krause examined some forty specimens, obtained in the course of a few weeks from cases of fungous arthritis in Prof. Volkmann's Clinic Halle, and found the bacilli in all. The examination of twenty sections taken from the wall of a cold abscess resulted in the discovery of but two bacilli. Kanzler reports the finding of the germs in eight out of fifteen specimens of bones and joints. Castro Soffia, who examined many specimens of tuberculosis in bone, always found the bacilli.

2. A second proof of this point rests in the fact that in the majority of cases of tumor albus a distinct family history of tubercular or strumous disease can be elicited.

3. The proposition has been confirmed by experimental pathogenesis.

Schüller, of Greifswald, produced tuberculosis in dogs and guinea-pigs by injecting phthisical sputa into their lungs, and by forcing them to inhale solutions of sputum and tubercular detritus half an hour daily for several days. Subsequently, by wrenching and contusing the knee joint he succeeded in developing in most of the animals a fungous synovitis, sometimes attended with suppuration. Healthy animals, when subjected to the same lesions, did not develop the disease. Hütter showed that injections of tubercular matter (sputum, etc.) into the joints of animals caused this "fungous hyperplastic synovitis," as he terms it, as well as the fact that from this primary focus general tuberculosis may follow.

The insertion of fragments of granulation tissue from tumor albus of the human being into the interior chamber of a rabbit's eye produced tuberculosis of choroid, and, later, general tuberculosis. König caused general tuberculosis by the introduction of synovial fungosities into the general circulation of animals. Barwell doesn't agree with Schüller's conclusions, though the grounds of his unbelief are not convincing.

Very interesting experiments have been made by Müller, with a view of demonstrating the channel of infection.

1. Tubercular matter was injected into the femoral arteries of sixteen rabbits, without result.

2. Again, the injections were made into the crural arteries of ten rabbits (the nutrient arteries are branches of the crural), and tuberculosis of bone developed in some.

3. Finally, the injections were made into the nutrient arteries themselves. Twenty goats, besides dogs and sheep, were operated upon, and, later, most of the former exhibited typical tubercular foci in the epiphyses. One young goat began to limp at the end of four months, and in another in nine months his knee joint was in a condition of typical fungous inflammation. The joint disease was found to have been developed from a primary cheesy focus in the head of the bone, the focus containing a wedge-shaped bony sequestrum.

Sternberg, also, failed to produce tuberculosis in animals by injecting inorganic material into their circulation.

Such studies would seem to afford conclusive proof of the relationship of tuberculosis and tumor albus.

"But," one is tempted to say, "if *tumor albus* is the result of the irritating action of the *bacillus tuberculosis* upon the tissues in and about the joint, why is the disease not invariably fatal? Why do not the bacillar microbes emigrate, invade the membranes of the brain, the lungs, or the intestinal tract, and there do the deadly work which the experience of every physician teaches him they are capable of doing?" Well, that such unfortunate cases do occur is well known, but luckily the percentage is not so great as experiments of Villemin upon the lower animals would lead one to fear.

While the human being is easily inoculated with the tubercular virus, the germs obtain a general foothold only under circumstances exceptionally favorable.

We are all familiar with the appearance of those large masses which appear so frequently in the cervical region of so-called strumous individuals, and represent aggregations of enlarged, cheesy lymph-nodes. We know from personal experience, perhaps, that such subjects may attain an advanced age, whether these soft, cheesy deposits discharge externally, gravitate through the inter-muscular planes of the neck to the subcutaneous tissue of the sternal region, or become absorbed. And yet the fact that the *bacillus tuberculosis* is the cause of this disease also has been demonstrated by Schüppel and many others. The germs are found in the diseased glands.

In like manner severe *scrofulous ozena*, many rectal fistulæ, certain marked ulcerations in the pharynx and soft palate, as well as some ulcerative conditions of the skin allied to lupus, are due to miliary tuberculosis; but the patients do not necessarily perish from the generalization of the specific disease.

A diminished vitality of the tissues is probably a prerequisite condition whether due to traumatism or not. Then we can think of the cells as unable to ward off the blows of the vegetable parasite, either from individual weakness or from the fact that the excretions of normal cell-metabolism are changed and no longer antagonize the growth of the microbe.

Statistics, however, prove that from the time the tubercular bacilli effect a lodgment in the system, the danger-signal is constantly flying. Such people are "suspects," as Volkmann has put it. The initial ravages of the invasion may be apparently effaced and the patient fully restored; yet it appears probable that some germs are still lurking in the old focus, are in a resting state, and only await a favorable accident that they may renew their work of destruction.

Esmarch, König, Caumont, and others, are agreed that amputation and resection, though beneficial in their immediate results, offer but little in the way of eradicating the tubercular taint, fifty per cent. and over of their cases dying subsequently from the effects of the disseminated tubercular virus.

Conclusions:

1. Tumor albus is purely the result of local tuberculosis of joints.

2. The bacilli lodge in the majority of cases in the extremities of the bones and usually on the distal side of the epiphyseal cartilage; though the synovial membrane may be the point of primary infection.

3. The primary osseous foci may remain latent for years, or may never infect the adjacent joint, but these are exceptional cases. The danger of infection increases with the softening of the cheesy focal deposits.

4. Preceding the specific infection of the joint, a diffuse arthritis of an innocent type may ensue from the mere proximity of the focal disturbances.

5. Healing may ensue at any stage; but, while joint-functions may be restored, anchylosis with or without luxation usually takes place.

6. In a large number of cases, systemic infection follows tumor albus, whether operative treatment be resorted to or not.

Dr. E. A. Smith read the second paper, on the

### SYMPTOMATOLOGY AND DIAGNOSIS OF TUMOR ALBUS.

It is extremely difficult to discuss the symptomatology and diagnosis of tumor albus, without treading occasionally upon ground so ably covered by the preceding speaker. Indeed, it may be said, that to leave out all discussion of a pathological nature, would render my part of this evening's proceedings a mere enumeration of points in differential diagnosis.

At the outset, it is understood that modern pathology applies the name tumor albus, or in common parlance, "white swelling," to all chronic joint inflammations due to tubercular infection, and thus tumor albus becomes synonymous with strumous or tubercular arthritis. In the next place a close study of joints attacked by tubercular inflammation reveals a common pathological process, varying only with the tissue first involved and the character of the destructive change. There is, in consequence, a common symptomatology, modified only by the anatomical peculiarities of the joint attacked and the stage of the pathological process. Hip-joint disease, for instance, is still treated by many writers as a distinct affection, although it is only a tumor albus obtaining its importance from its frequency, and the special symptoms due to the situation and functions of the joint.

Avoiding, then, an enumeration of the symptoms connected with each joint, we can proceed to a more philosophical study of them, as they are found in tubercular inflammations of the larger joints, reserving a few words, suited to the limited time allowed each speaker, for special reference to certain joints.

In attempting to classify cases as to their widely-varying clinical features, it is difficult to do so on the basis of their pathology as regards the point of origin and progress of the affection. Modern authors admit that tubercular arthritis starts most often as an ostitis, but they are also agreed that in the early stages, a differential diagnosis between tubercular arthritis starting in the synovial membrane and the same inflammation starting in bone is usually impossible.

There is, however, a notable difference in the clinical course of cases, depending upon the early or late occurrence of suppuration, and we may, with advantage, first dispose of the symptomatology of tumor albus up to the time of distinct evidence of suppuration. During this time the pathological process is in the inflammatory stage, and the cases are such as are seen in private practice, as compared to older cases with fistulæ, sinuses, and other unmistakable evidence of marked destructive changes which present themselves at hospital clinics for operations of last resort.

Leaving for later discussion the significance of the constitutional condition of the patient, we find in cases of tumor albus a history of joint trouble of long standing, with comparatively few subjective symptoms, but many pronounced objective evidences of disease, properly called signs.

Taking up the symptoms first, pain is, perhaps, most prominent. It varies in severity with the amount of inflammation and the character of the patient, but early in the disease it is always worse upon moving the joint after a period of rest. Upon rising in the morning, the joint is more tender than after some use. Later, when congestion gives place to inflammation and exudation, the pain is more pronounced when the joint is in use. A sharp, shooting pain, awakening the patient when asleep, or suddenly occurring when the affected limb is at rest, takes the name of "starting pain." Early in the disease sleeping children frequently wake with a cry, or, as is often noticed, only partly awaken, scream sharply, and sink off into deep sleep again. This is due to the fact that during sleep the muscles are relieved, and upon some slight motion of the inflamed structure in the articulation, a reflex spasm is produced which violently moves the sensitive parts and causes sudden agonizing pain. Another peculiarity of pain in tumor albus, is the so-called "sympathetic" or referred pain, notably a feature of hip-joint disease. Owing to irritation by the pathological process in the course of a nerve, painful sensations are referred to its terminal filaments. Thus, in hip-joint disease, pains about the inner side of the knee are symptomatic of irritation of the obturator nerve in the neighborhood of the hip-joint. In children it is well to remember that pain may be denied when actually felt from fear of some operative procedure, but a sudden jar or wrench of the affected joint will elicit an instinctive cry. When a patient complains of a dull, constant, boring pain with occasional exacerbation, and there exist points of extreme tenderness upon pressure over the articular ends of the bones, it is strong proof that the disease started in the bone ends.

With the early twinges of pain the patient learns to guard the joint against full extension or flexion, and this soon becoming automatic, produces a slight stiffness of the joint in the upper extremity, and a limp in the lower. As the inflammation progresses this pseudo-anchylosis does also, and the joint tends more and more to remain in the position of maximum relaxation midway between flexion and extension. Up to the time when degeneration changes become marked, this pseudo-anchylosis is merely reflex muscular spasm, and under anesthesia free movement of the joint can be obtained. While the function of the joint is thus slowly impaired, there is also a loss of muscular power owing to lack of exercise, and the patient soon begins to notice a wasting of the muscles of the affected limb.

In the course of time, the pathological process carries the disease further, and these subjective evidences of trouble give place to distinct objective features. An examination usually discloses a notable increase of temperature in the affected joint; tenderness on pressure, and pain on motion are pronounced, and the function of the joint is steadily more impaired. In the lower extremity limping and stiffness have, perhaps, increased to inability to support the body, and a cane or crutch is necessary. In the upper extremity the arm is kept at rest in its semiflexed position, and the patient uses the other arm and hand in all movements, including the commonest motions, such as carrying food to the mouth.

Examination also reveals the joint seemingly much enlarged, but careful measuring in many cases shows the enlargement to be apparent and not real, especially in those cases which originate in the bone ends, and where the consequent synovitis and effusion are minor features. The joint appears relatively large when compared with its fellow, because the bony parts composing it maintain their usual size, while muscular atrophy may have so diminished the limb above and below the joint, that the measurements are one to three inches less than in the healthy limb.

But when the synovitis is primary or has become pronounced secondary to osseous trouble, the joint itself enlarges. Synovial effusion first uniformly fills the capsule, obliterating the normal depressions, and then distends it. In the knee-joint, for instance, the patella is floated and the swelling has a distinctive quadrilateral form. Here the tape line shows marked enlargement of the joint, and palpation reveals the presence of fluid; or, if the condition in the joint is rather pulpy degeneration than serious effusion, the boggy feel of false fluctuation is present.

In either class of cases the patient is anemic, and the apparent or real swelling is white and shiny, from which appearance the disease received its name, tumor albus. The swelling is also tense and smooth, depending on the amount of effusion, and the superficial veins are large and prominent, due to the obstruction to the return flow in the deeper vessels. By this time the inflammatory action has infiltrated and softened the joint, capsule, and ligaments, and, if the articulating bones are grasped and moved upon each other, unnatural motions can be produced, notably a "lateral motion" of ginglymoid joints. Lastly, in the picture of severe tumor albus, before the advent of suppurative changes, the tonic muscular spasm fixing the articulation may be replaced by neoplastic formation of fibrous bands, which cause a more or less complete anchylosis that anesthesia cannot relax.

The pathological changes which produce the foregoing symptoms and signs may consume several months to one or more years. On the other hand the disease may proceed rapidly to suppuration and complete disorganization of the joint, or reach the same end by long continued suppuration locally, and the development of hectic fever and septicemia as constitutional conditions. The origin and special significance of suppuration must be discussed by another essayist, and it must suffice here to say that its occurrence heralds the onset and accompanies the course of progressive destruction. Its early detection, therefore, is important as regards prognosis and treatment.

With the suppurative process, lymphangitis and glandular enlargement in the neighborhood of the affected joint become more noticeable than in tumor albus before suppuration, in which latter class of cases lymphadenitis, being merely an evidence of excess of inflammatory absorption, may be so slight that glandular enlargement cannot be detected. The simplest cases of tumor albus with suppuration are those in which the inflammatory exudate breaks down in the particular structures. Here the signs and symptoms of acute or cold abscess are added to the features of strumous arthritis. Pus formation in the bone ends may continue some time before detection. Chills and rise of temperature, especially after an exacerbation of inflammation, excite suspicion, and this becomes certainty if over the bone ends an excessively tender spot, with fluctuation, can be found. If the purulent degeneration begins in the joint cavity, a condition of pyarthrosis soon develops, and, if the process is acute, fluctuation, chills and fever make possible an easy diagnosis of suppurative degeneration. When, however, the granulation tissue is abundant, absorption slow, and there may be no chill nor marked fever, still the temperature may be noticed to ascend each evening one or two degrees, and to fall to normal again each morning. Here the introduction of an aspirating needle may give positive assurance of the presence of pus.

There are still many cases in which suppuration is slow, with a tendency to caseation, in other words, lingering cases accompanied by formation of cold abscesses. They may remain stationary for long periods, or caseation may be followed by firm fibrous or bony anchylosis and subsidence of the virulent powers of the tubercular bacillus. In these cases there are no new symptoms, but an amelioration of those already described, excepting anchylosis, which remains as an evidence of what has taken place. These slow cases, however, are liable to awaken to new activity, and then they follow the road which most of these lingering cases do, and ultimately the pus burrows to the surface, usually in the close neighborhood of the joint, but sometimes far removed.

This pathological departure gives origin to the easily recognized fistulous tracks leading to the diseased joint, or to sinus tracks leading to carious bone, and the surgeon takes advantage of these well-known signs of tumor albus to probe the seat of disease and verify by touch what his judgment has already diagnosticated. With the disorganized tissues thus within reach, he may push his inquiry further and microscopically examine for the inflammatory neoplasia due to and containing the tubercle bacillus.

Cases of tubercular arthritis which have reached this point are the ones usually presented at hospital clinics. Corresponding to their advanced destructive changes are exaggeration of the symptoms and signs already mentioned. Function is lost, or nearly so ; the form of the joint is still more abnormal, even to the extent of subluxation or complete dislocation ; lateral motion is easily obtained, unless bony anchylosis has developed; and, with lateral motion, is felt the peculiar grating arising from rubbing of exposed roughened bone ends ; finally, the joint is fixed in a more or less flexed position, depending on its position and the strength of the flexor muscles which influence it.

In conclusion, How are the symptoms thus spoken of in general to be applied in detail to make a diagnosis ? The hip and knee joints, owing to their size and importance, have some symptoms peculiar to themselves when affected by tubercular inflammation, but a knowledge of the common features of tumor albus will enable the surgeon to decide a question of differential diagnosis in any joint. Few cases are so typical as to present all the features mentioned, but in a case suspected to be tubercular arthritis, the presence of some of the before-mentioned symptoms and signs, with the additional features to be elicited by the judicious use of tape line, aspirating needle, probe and microscope, render a diagnosis reasonably sure. A study of etiology, covering family history, traumatic influence, age of patient, duration of the disease, and coincident presence of systemic tuberculosis, may fix the diagnosis beyond any reasonable doubt.

There is, besides, the question of differential diagnosis between tumor albus starting in the bone and that starting in the synovial membrane. Upon this differential diagnosis in the early stages depends the prognosis as to the necessity for arthrectomy or resection, or the combination of both operations, although it may be said that operative treatment usually begins as an exploratory incision, and progresses according to the nature of the pathological changes that are found. Cases are probably synovial in origin when there is actual and early enlargement of the joint due to synovitis and abundant synovial effusion. Lateral motion is present, but not accompanied with grating of denuded bone surfaces. On the other hand, cases which arise in bone have deep, boring pain with enlargement and tenderness in one or more of the bone ends composing the articulation, while the joint itself is not enlarged and does not exhibit lateral motion. Muscular spasm and atrophy, and the other signs and symptoms of tumor albus are, of course, common to both classes of cases.

Dr. JOHN PARMENTER read the third paper, on

### THE TREATMENT OF TUMOR ALBUS.

Acute diseases, as we all know, tend to recovery, and often in spite of the misdirected and blind efforts of the physician or surgeon. With chronic diseases, however, it is different. Nature has the underhand, so to speak, and oftentimes all the resources of the medical attendant are taxed to restore her to the supremacy. In treating a disease like tumor albus, therefore, we should carefully ask ourselves: firstly, Upon what principles should we base our treatment? and, secondly, How can we carry them out most safely and efficiently. I think we are all agreed that the two principles upon which we base our therapeutic measures against this disease are improvement of the general health and rest of the diseased part, and how we can best carry out these principles forms the subject of this paper to-night. I can only hope to give the outlines of the treatment applicable to tumor albus, but I trust that in giving these I may make them sufficiently clear and give due emphasis to that which I consider of most importance.

The treatment of tumor albus, therefore, naturally divides itself into the constitutional and local. Sometimes the former alone suffices; usually, however, a combination of the two is necessary. First of all, then, how can we improve the general health of the patient and overcome his predisposition to this form of trouble?

So much has been written on the constitutional therapy of tuberculous disease, especially of phthisis, that it is somewhat strange that surgeons, as a class, do not better grasp the details of the same. Certain it is that the average surgical text-book dismisses this side of the question with a few general remarks, and, perhaps, this explains why many surgeons attach relatively so little importance to constitutional measures, and proceed so readily and prematurely to operative means. To be sure, the incapacity of the patient to get around when one of the larger joints (as is too often the case) is involved, seriously hampers the surgeon. For the very reason, however, that the patient is thus denied exercise in open air and sunshine, the surgeon should develop to their utmost limit of utility the measures which remain to him. When the patient can be equipped with appliances which will permit exercise out of doors, we have made one long step toward bettering his general condition. Exercise to the point of tire in ordinary weather, avoiding only the very unfavorable, is of great therapeutic value, direct evidence of which is to be seen in the usual deterioration of health which these cases suffer in the winter time, when exercise is often much interfered with. If I see a patient go through a winter without any change for the worse, I prognosticate recovery, and have as yet to see the case where such prognostication proved incorrect. The explanation naturally lies in the fact that a patient who can dispense wholly or in part with so important a feature of his treatment and still improve, or at least not retrograde, must be in a condition which gives good promise of future improvement. By all means, then, exercise in the open air when possible. But suppose our patient cannot get out, at least for the time being. Then massage should come to our aid. This should be entrusted to some member of the family, previously instructed by the surgeon as to its method of application, or, better still, to the professional masseur when possible. The room of the patient should be thoroughly aired, in winter time never less than three times a day, for fifteen minutes at a time, the patient being sufficiently covered and then uncovered by degrees as the room becomes warmer. He should also, during this time, be instructed to take deep inspirations up to the point of tire, increasing the number gradually on each succeeding day. He should have a bath (sponge or other) if possible. The addition of a little alcohol or sea salt to the water is advantageous. If no spinal or other disease contra-indicate, at the conclusion of the bath a sponge full of water should be placed upon the back of the neck and squeezed, so that the water may run down the spine. The water should be gradually made colder on succeeding days, until tolerance of cold water is established. This should never be done more than once at one sitting, and should be followed by gentle friction with a dry and crash towel. Sea air is a powerful adjuvant to our therapeutic measures. Food should be liberal and nutritious. Let the customary meals be regular. The mid-day meal (if luncheon) should be just as large as the dinner. The dinner should never be later than 7 P. M. Give food between meals, preferably in the form of milk. A glass of hot milk early in the morning is very grateful to some. Where milk disagrees, koumiss makes an efficient and agreeable substitute. Maltine and milk together are also good. Milk or some substitute should also be taken before bed-time, and during the night should the patient be awake.

In the medicinal treatment of this condition, Barwell recognizes two clinical types, one the delicate, thin skinned type, the other that with thick connective tissues. To the former he would give iron, quinine, maltine, and cod liver oil. (Iodine in these cases must be used sparingly and cautiously and with other remedies.) To the latter he gives iodine, mineral acids, vegetable bitters and occasional purging. Here he regards cod liver oil as useless. I believe such a division to be useful, and borne out by the experience of most surgeons; but it must be remembered that many (perhaps the majority) of the cases do not fit exactly under one or the other types, but combine the two, and that in these cases a combination of the modes of treatment is better than either one alone. Such, at least, has been my own experience. Whatever the kind of case one rule holds good, "take care of the stomach." The observance of this rule by physician and patient has brought success in many cases. In no condition is strict attention to the primæ viæ and, especially the stomach, of more importance than in the scrofulous or tuberculous. Where acids seem indicated, I am fond of the following combination, recommended by Beale, for indigestion:

| | |
|---|---|
| ℞—Spirits Chloroform, | ℨ iii. |
| Acid. Nitro-muratic. dil. | ℨ vii. |
| Syrup. Limonis. | |
| Tinct. Aurantii | aa ℨ xi. |

Sig. Teaspoonful in water twenty minutes before meals.

Oftentimes acids are better borne after meals, especially if there be any intestinal indigestion. When gastric catarrh is present, carbonate of bismuth often acts well.

To promote the appetite I use the following mixture, recommended to me by Prof. Roberts, of University College, London, and to his loud praises of its virtues I can heartily add my own.

It runs thus: Acid hydrocyan dil. min. iii., Sodii Bicarb grains x., Infus. Gentian, ℥i. The whole to be taken at one dose, and ten minutes before dinner and supper. The infusion closely resembles that of the British Pharmacopeia and is made as follows:

| | |
|---|---|
| ℞—Radicis Gentian. incis. | ℥ ss. |
| Cortic. Limonis (fresh) | ℨ iss. |
| Cortic. Aurantii | grs. xlv. |
| Aquæ Bullient | oii. |

Let this infuse for one hour, then strain and put in a cool place. Containing as it does no alcohol it decomposes rather readily and should be made in comparatively small quantities at a time.

A quite extensive experience with this combination leads me to prefer it to any other known to me, and furthermore it can be given coincidently with the next drug to be mentioned, viz.: Cod liver oil. The pale oil is preferable to the others, and, though theoretically best given an hour after meals, is often better borne when taken directly after the same. Two to four teaspoonfuls after breakfast and the midday meal, agrees usually better than when one of the doses comes at night. Some, however, can take cod liver oil only at night. Disagreeable at first, the majority of patients soon learn to like it, and the taste once acquired, usually lasts for a long time, a remark which does not maintain with emulsions. Cod liver oil may often be given advantageously with tonics, with acids, with pancreatine and so on, according to the indications. When it disagrees change the time and vehicle. As a rule the gentian mixture above mentioned, before meals, and the cod liver oil after meals, make an efficient and not unpleasant way of employing these remedies.

Iron, arsenic, nux vomica, and other tonics all have their place according to the individual necessities of the case, but should never be used indiscriminately. Should alcohol ever be given? Ordinarily, I believe it can be given with advantage, but only with the two principal meals of the day. However, where moral or other considerations make its use undesirable, it can be easily dispensed with. Malt liquors are preferable, after which, in the order of merit, come the red wines.

The limits of this paper prohibit further remarks upon the treatment of the constitutional condition associated with and underlying tumor albus. I have only been able to hint at the methods which have stood me in good stead in dealing with this disease. Such a plan can be adopted with advantage in the majority of cases, rich and poor alike. The poor have rather been kept in mind than the rich, so that any advice given may be applicable in all cases. Drugs I deem of least importance, but still valuable, and so have been led to mention the best known and most efficient, giving at the same time a hint or two as to their exhibition.

At the same time that the physical condition is being carefully attended to, tumor albus requires local treatment. A brief inquiry into the recognized methods of the same, forms the second portion of this paper. Bearing in mind again that the principle underlying all local treatment is rest, let us see how we can best attain this end.

In tumor albus of the upper extremities, where the outdoor life of the patient is not interfered with, local treatment is often quite subsidiary. With the lower extremities, however, it is quite different. In the latter cases we must have a method which will give the affected part complete rest and at the same time permit the patient to get about if the inflammatory condition be not too acute to contra-indicate the same. Naturally our apparatus must vary with the joint affected, and this is not the place to go into therapy of the separate joints. I shall content myself, therefore, with merely mentioning for the hip, extension and a plaster-of-Paris bandage, fixing pelvis and thigh, the latter to be used when all deformity has been overcome, and the former to be discontinued at the same time. The Taylor splint is much used by surgeons, but is costly and permits deformity in a position of adduction with extraordinary ease.

For the knee the Thomas or Taylor splint is perhaps the best means we have of immobilizing this joint. Their price, however, forbids their use by the poor, in which case we can substitute an apparatus made of felt, soaked in a solution of shellac and allowed to harden on the patient. This is inexpensive and forms a very fair substitute for the above-mentioned splints. The ankle can be immobilized with plaster-of-Paris, water-glass or other means, and a high shoe put upon the opposite foot.

Beside immobilization, what else can we do? We can make (1) external applications; (2) injections; (3) ignipuncture; (4) incision and drainage, erasion, exsection or amputation.

Among external applications we can include absorbing ointments, water, with salt or carbolic acid in the form of poultices continuously applied, ice bags, irritating agents, as the moxa, cautery, fly blisters, and the like. Of all these agents, probably flying blisters and tincture of iodine are most efficient, and least likely to be followed by injurious results. It is hard to imagine how external applications can be of use in a disease affecting tissues so deeply situated. Where good results have followed from their use I am inclined to think that they were due to other measures used coincidently, and I, therefore, dismiss this method of treatment without further mention.

Chief among the injections are carbolic acid, iodoform-ether, iodoform-oil, and balsam of Peru.

Hütter was the first, I believe, to recommend the injection of a two-three per cent. carbolic acid solution by means of a Pravaz or other suitable syringe. The fluid is injected into the para-articular tissues with a moderate amount of force. Properly done, so far as I know, this never does harm, but very often is disappointing. Testimony as to its value is very conflicting. However, the good results attained by Schede, of Hamburg, some of which I personally observed, should lead us to give these injections further trial. Strict antiseptic precautions are to be observed and the injection repeated every eight or ten days.

In 1885, Verneuil published an article entitled, Injections of Iodoform-Ether into Cold Abscesses, claiming for them, among other things, that they were innocuous. Since that time many observers have used his method with the result of disproving his assertion. Among the consequences following their use frequently occur severe burning pains, continued vomiting, narcosis lasting over several hours, and most undesirable of all, stretching with gangrene of the abscess wall, due to the rapid vaporization of the ether. In the face of such experiences as these, I think we must look upon iodoform-ether as an injection to be used with great caution, and never in extensive abscess cavities. It is given in five per cent. solution, anywhere from 10-100 grammes being injected at a time, according to the necessities of the case.

Iodoform and olive oil in the proportion of 1 to 5, of which 2 to 3 c.cm. are injected every eight days, makes a combination quite free from the objections just noted, when ether replaces the olive oil. Under its use giant cells cease to grow, the abscess contents lose their infecting power, and tubercles, together with tuberculous granulations, disappear. Pain becomes less, very early swelling diminishes, and the part becomes harder and firmer. Even abscesses become smaller and finally disappear, and the mobility of the joint up to a certain point returns. Such is the course pursued by the favorable cases which unfortunately are only too rare. Many are improved, but not cured, and many ultimately require operation. The oil should be sterilized and the iodoform mixed with it first before use. A brownish-red color indicates the formation of iodine which rapidly occurs. The syringe should be emptied with considerable force in order that the solution may come into contact with all the diseased tissues. A sublimate gauze bandage completes the procedure.

Balsam of Peru has been highly commended by Landerer and by VanVamossy, the latter, however, previously cutting away all diseased tissue. The good effects are identical with those of iodoform-oil. As used by VanVamossy, albuminuria was frequent, owing, undoubtedly, to the fresh wound surfaces with which it came in contact.

In very, very few cases do injections effect a cure ; only one-third of the cases so treated show improvement. We cannot, therefore, regard them as potent agents for good in treating tumor albus, probably, because all the diseased parts are not and cannot be reached by them.

Another method of procedure is that by ignipuncture, which consists in applying the actual cautery to the interior of the bone substance. Introduced by Richet and Kocher, independently of each other, it has been widely used on the continent. To Park, of this city, belongs the credit of being the first surgeon to recommend and practice ignipuncture in America, and concerning the indications for and the technique of its use I quote from his excellent brochure on Tuberculosis of Bones and Joints, and Its Treatment by Ignipuncture, published in the *Medical News*, August 30, 1884:

In general terms we may hold that ignipuncture is indicated in all cases of chronic ostitis where fluctuation and sinuses or other indications of points of softening and breaking down do not call for the curette or chisel, and in many cases when after removal of products of softening, the cautery can still be used to advantage to stimulate surrounding bone into a normal activity. Its application may be intra-osteal or intra-capsular.

In the former, the glowing platinum is either plunged through the skin and soft tissue right down to the bone and into its substance, or else an incision is made through a part or the whole of the soft parts, since it is not so much disturbance of these latter which is primarily aimed at. Antiseptic precautions and dressings are of course indicated, and if the incision has been a large one, it may be partially closed, but ample opportunity must be given for drainage, so long as it may be necessary. When combined with other operative measures, a depot of softening may be cut down upon, its contents removed, the sharp spoon used as indicated, and from this bone cavity the cautery point may be made to perforate in different directions. The bone exposed to intense heat is burnt into a crust; around this takes place an active inflammation which leads first to an osteo-porosis, and later to an osteo-sclerosis, which means virtual recovery. As I have already tried to show how often a primary bone lesion leads to a serious joint complication, it will be seen that the intra-osteal ignipuncture may have a most important prophylactic effect as against the same; in fact, my own experience has taught me its value in this respect.

Intra-capsular ignipuncture may be made either with or without previously opening the joint. If the joint is supposed to be in a fungous condition, it should certainly be opened and cleaned out, but if it seems to be merely a lesion in a bone-end lying so close to the joint surface that it can only be approached by going through the joint, then no hesitation need be felt in burning right through the capsule and its cavity into the bone; only being doubly careful about the antiseptic precautions. Lücke does not hesitate to lay the joint freely open. If the joint surface is healthy, and only bone is diseased, joint re-action is unlikely to occur. This measure is also said to be serviceable in chronic rheumatic and other forms of synovitis, though I have had no personal experience with it in such cases.

In every case in which I have resorted to it, I have administered an anesthetic, though Kocher states that he seldom gives one. The actual cautery used in this way, and with the avowed purpose of securing, not a derivative effect, whatever that may be, but a speedy and acute reactionary inflammation, is to be considered quite apart from the vigorous counter-irritation which so many, Syme, for instance, have sought to induce by the moxa or any external application of cautery or issue. . . . So far as my own experience goes, the first result of its use is a speedy relief of the characteristic ostitic pains; tenderness on pressure disappears later.

Lastly, we can treat tumor albus by incision and drainage, erasion, exsection, and amputation. Inasmuch as our application of any of these methods of procedure must vary with the special part affected, and a description of them all is incompatible with the limits of this paper, I shall content myself with a brief consideration of the indications for such radical operative measures—a matter far more difficult to learn than the technique of the operation. I regard one or the other of these procedures indicated in all cases where (1) an abscess exists; (2) there is positive bone disease; and (3) cases that have not improved under the long-continued and persistent use of the milder and more conservative methods of treatment.

Incision and drainage in cases of abscess formation are, in my opinion, of doubtful utility. I believe it is a good rule in the great proportion of cases never to open the abscess unless you are ready to make a radical operation. Spontaneous discharge of the purulent collection causes less disturbance to the patient usually than opening with the knife, no matter how rigid the antisepsis. The above remarks naturally do not apply to cases of acute infection. Here there is rapid disorganization going on, with severe symptoms, and speedy incision is urgently demanded.

Erasion, or arthrectomy, in the proper sense of the word, is applicable to those cases in which the synovial membrane and ligaments only are diseased and removed. With those who look upon every case of tumor albus as originating primarily in the synovial membrane, it is a favorite operation. The removal of a certain amount of bone, or cartilage, when diseased, is permissable. Erasion, therefore, is indicated only when the disease is chiefly synovial, and in such cases it possesses advantages of signal value, among which are no shortening of limbs, no deformity, and no arrest of growth from interference with the epiphyses.

If the condition has progressed so far that both bony and soft parts are much involved, and yet there remains sufficient sound tissue to warrant it, exsection should be made. It may be partial or total, but in either instance two things are imperative—to remove everything diseased, and to avoid removing anything not diseased. Observance of this rule, with careful cleansing of the joint cavity, are essential elements of successful exsections.

Amputation is to be used only when other methods are unavailing. How much and when to exsect, or when to amputate, will depend upon the necessities of each individual case, and naturally the training and experience of the surgeon, in deciding this question, will be valuable in proportion as they have been thorough and extensive. No more perplexing problem presents itself to him than this very one, and therefore every case demands his earnest and thoughtful consideration. Without doubt, many surgeons are too radical, and cut away much that would be of immense use in the future. Especially is this true in the case of children, upon whom, in my opinion, radical measures are far too often pursued. Conservative treatment here will do wonders in cases that seem almost hopeless. A more thorough recognition of the value of constitutional measures and a more perfect immobilization, by which the lower limbs may imitate in their mechanical surroundings the upper limbs, will make resort to the knife more and more infrequent. I have three times seen general tuberculosis follow exsection of the hip. This is best explainable on the ground that infection of the fresh wound surfaces took place, the bacilli thus readily gaining access to the general circulation.

At the same time I do not wish to be understood as a procrastinator. There is undoubtedly in every case a time beyond which it is wrong to delay; but when the surgeon thinks the time for operation has come, he should have the consciousness of having given all the less radical measures a faithful and thorough trial. When can a patient be regarded as cured? So many patients, after operation, die of tuberculous trouble, either recurring in the same place or somewhere else, that it is desirable, for purposes of prognostication, to have some time limit, arbitrary though it be. To this end Dr. Schmid-Monnard, of Halle, has collected statistics, which he has given in the *Centralblatt für Chirurgie*, No. 52, 1889. He has collected 116 fatal cases which were under observation after resection from two and one-quarter to fourteen years, with the following result: Seventy-four (sixty-four per cent.) died within one year of the day of operation, nineteen (sixteen per cent.) died within two years, leaving twenty-three (twenty per cent.) living after two years, of which the majority died from two and one-half to three and one-half years post resectionem. He says: "After the expiration of two and one-quarter years, probably only four per cent. of all cases resected die from their disease or the immediate consequences of the same." He thinks that all who are healed after two and one-quarter years will probably remain so, and that resection statistics should not include cases observed for a less period than this. This, I think, agrees in the main with the experience of most surgeons. To be sure, there will be exceptions, but the majority of cases are doubtless within the rule.

In conclusion, let me say that no attempt has been made to present a formal paper on the treatment of tumor albus. I have simply tried to hint in a familiar way at the methods usually employed in treating this disease, and to emphasize some points which the experience of others and my own have found useful.

Dr. W. H. Bergtold opened the discussion. He expressed his appreciation of the honor of being called upon to lead the discussion, and said the subject had been so thoroughly covered that he could find but little to add. One phase of this question is of more than passing interest, to-wit: the fact that tubercular arthritis is so common in children. Given a case of *tumor albus*, the chances will probably be fifty per cent. that it is in the extremity of a child or young person.

The tubercular infection may be inherited or acquired; while the first is, as yet, not accepted by all as proven, still the experiments of Landouzy and Martin (Ref. Haulb., vol. viii.—p. 340) seem to show that tuberculosis can be transmitted from parent to fetus. Be this as it may, and starting with a given infection, why is it that the manifestations are exhibited, as a rule, in the lungs, in an adult, and in the long bones and their joints, in a child? It looks reasonable to assume that those parts which receive proportionately the greatest amount of blood, and at the same time have the slowest and weakest drainage by the lymphatics, will receive the most infectious material, retain it the longest, and first show signs of the tubercular disease.

These conditions are abundantly present in the ends of the long bones of the child; the epiphysis has sent to it a goodly share of blood, both because of great activity of growth, and ossification, and the great pressure received in play, romping, etc., incidental to a child's life. In the adult, these parts have matured, and while still receiving, in comparison, a larger share of blood than the other portions of the bone, yet the amount is not nearly so great as that taken by other parts of the body, the lungs, for example. In acquired tuberculosis, probably ninety-nine per cent. occur through the lungs and intestines. In each place lymphatic absorption is active, and all of the infectious material is probably sifted out by the lymph nodes, leaving the lymph innocuous, and starting a tubercular focus in the node. In congenital tuberculosis, however, infectious material may be circulating, or remaining dormant until a predisposing cause, as an attack of measles, etc., so lowers tissue resistance that, with the epiphyseal supply of blood (being relatively large), sufficient infectious matter is received, and retained (by virtue of slow lymphatic drainage) to inaugurate the tuberculosis in the form of an ostitis, and later, an arthritis. If this view of the case be correct, it is easy to see why the ends of long bones, and their joints should suffer from tuberculosis in children, so often in preference to the lungs or other parts of the body. Such an idea is manifestly open to many criticisms, but it appears to accord quite well with the physiology of the parts and clinical manifestations.

Dr. Roswell Park spoke next. He recalled his experience before the city Society some years ago, when he read a paper presenting what were then new views on this subject. He was then derided, and, partly to defend the position he then took, he has since examined microscopically his cases of tumor albus. In many, but not in all, he found the tubercle bacillus. He has not seen so much secondary dissemination as is claimed by German authorities, and he thinks it is owing to the better condition of our working classes compared to those of older countries, especially Bohemia and Ireland. He believes no surgeon in Buffalo would obtain the mortality rate of sixty-four per cent. from secondary dissemination claimed by German surgeons after operations for tumor albus.

Dr. McCHESNEY related the history of a case of tumor albus. The patient had a family history of tuberculosis and a personal history of traumatism, from which the disease dated. The case improved for a time under treatment, but, after passing into other hands, died of secondary phthisis pulmonalis. Dr. McChesney thought more should be done to prevent milk of tubercular cows from being used as a food.

Under Presentation of Specimens, Dr. Wm. C. Krauss reported the following case and presented part of the thoracic aorta with a ruptured aneurism, which had produced sudden death.

The history of the case is as follows:

The patient, a young, well-built man of twenty-four years, had for some time complained of neuralgic pains over the right hypochondrium, and a cough, spasmodic in character, which had resisted all treatment. Being confined to the house on account of no work, he had occasion to step to the window to see a passing funeral. He returned to near the stove, sat down, and fell forward—dead.

The case was reported to the proper authorities, and at first it was thought unnecessary to hold an autopsy, as "in all probability it was a case of heart disease." The autopsy, however, revealed a different state of things. On opening the thorax, the right general cavity was found filled with a light straw-colored fluid, which on removing changed to sero-hemorrhage, and finally nothing but huge blood clots remained in the floor of the cavity. The right lung was collapsed and retracted. The causa mortis was decided at this point on receiving the information that the young man had contracted syphilis 18 months previous, and had allowed it to go on without treatment. The heart showed signs of fatty degeneration and infiltration. The arch of the aorta contained three quite large, soft, spongy, gummatous plaques. At about the middle of the thoracic aorta, a sacculated aneurism about the size of a pigeon's egg was found, which contained some blood clots, and with walls intact. At the lower part of the aorta was situated another sacculated aneurism, about the size of a hen's egg.

This aneurism was facing the right, lying upon the dorsal vertebra and infringing upon the diaphragm. Its walls were irregular and presented quite a large opening, through which the fatal hemorrhage took place.

The symptoms during life were easily accounted for by the pressure of the aneurism upon the intercostal nerves and upon the diaphragm, or pleuric nerve. The cause of the aneurism at such an early age was no doubt due to syphilitic infection.

